# Cardiovascular system and coronavirus disease-2019 (COVID-19): mutual injuries and unexpected outcomes

**DOI:** 10.1186/s43044-021-00202-4

**Published:** 2021-09-03

**Authors:** Eman Sobh, Muhammad Saad Reihan, Tamer M. S. Hifnawy, Khloud Gamal Abdelsalam, Sohaila Sabry Awad, Nehal Mostafa Hamed Mahmoud, Nariman A. Sindi, Hani A. Alhadrami

**Affiliations:** 1 Chest Diseases Department, Faculty of Medicine for Girls, Al-Azhar University, Cairo, Egypt; 2 Respiratory Therapy Department, College of Medical Rehabilitation Sciences, Taibah University, Medina, Saudi Arabia; 3 Cardiology Department, Faculty of Medicine, Al-Azhar University, Damietta, Egypt; 4 Alghad International College of Applied Medical Sciences, Jeddah, Saudi Arabia; 5 Public Health and Community Medicine Department, Faculty of Medicine, Beni-Suef University, Beni-Suef, Egypt; 6 Biochemistry Unit, Chemistry Department, Faculty of Science, Damanhour University, Damanhour, Egypt; 7 Independent Researcher, Bachelor Degree of Biochemistry, Faculty of Science, Cairo University, Cairo, Egypt; 8 Faculty of Pharmacy, Al-Azhar University, Cairo, Egypt; 9 Department of Medical Laboratory Technology, Faculty of Applied Medical Sciences, King Abdulaziz University, Jeddah, 21589 Saudi Arabia; 10 Special Infectious Agent Unit, King Fahd Medical Research Centre, King Abdulaziz University, Jeddah, 21589 Saudi Arabia

**Keywords:** Coronavirus disease, SARS-CoV-2, Cardiovascular dysfunction, Cardiovascular function, Public health implications

## Abstract

**Background:**

Cardiovascular system involvement in coronavirus disease-2019 (COVID-19) has gained great interest in the scientific community.

**Main body:**

Several studies reported increased morbidity and mortality among COVID-19 patients who had comorbidities, especially cardiovascular diseases like hypertension and acute coronary syndrome (ACS). COVID-19 may be associated with cardiovascular complications as arrhythmia, myocarditis, and thromboembolic events. We aimed to illustrate the interactions of COVID-19 disease and the cardiovascular system and the consequences on clinical decision as well as public health.

**Conclusions:**

COVID-19 has negative consequences on the cardiovascular system. A high index of suspicion should be present to avoid poor prognosis of those presenting with unusual presentation.

## Background

**Fig. 1:**
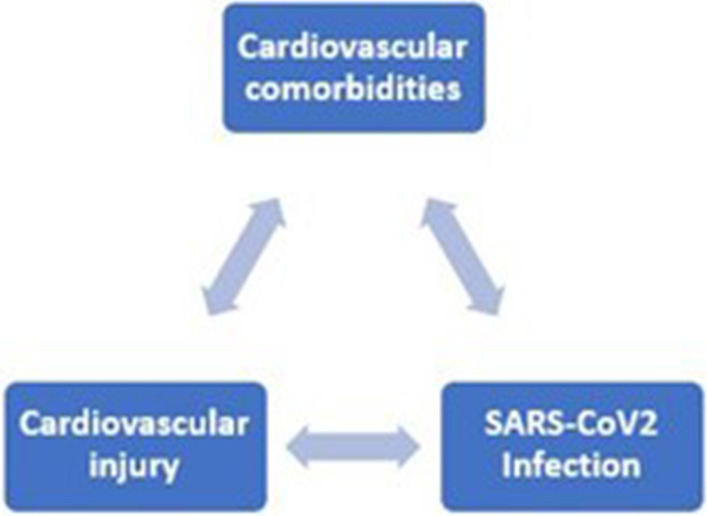
Mutual relationship between cardiovascular system and COVID-19. Cardiovascular comorbidities are linked to risk and mortality of COVID-19. At the meantime, COVID-19 is associated with cardiovascular injury and complications

Coronavirus disease-2019 pandemic is a major health problem that affected hundreds of countries and territories. It results in more than 200 million infected cases and more than four million deaths worldwide [1]. COVID-19 is caused by a novel strain of coronaviruses called severe acute respiratory syndrome coronavirus-2 (SARS-CoV-2) [2]. It is well established now that SARS-CoV2 has pulmonary as well as extrapulmonary effects [3], including renal, cardiac, nervous and gastrointestinal systems [3, 4]. Mutual interaction is present between COVID-19 and the cardiovascular system (Fig. 1). A link exists between cardiovascular (CV) comorbidities and both COVID-19 risk and outcome [5–7]. CV comorbidities were associated with disease severity and a greater risk of death [8, 9]. At the same time, COVID-19 was associated with several cardiovascular complications [10]. These cardiovascular complications include: venous thromboembolism (VTE), pulmonary embolism (PE), myocarditis, arrhythmias, ACS, and sudden cardiac arrest [9, 11, 12]. PE is the most devastating complication expected in severe COVID-19 disease [9]. In this review, we will illustrate the interaction between COVID-19 and the cardiovascular system, the mutual complications, and the impacts of COVID-19 on clinical decision-making and public health.

## Main text

### Pathophysiology of cardiovascular injury in COVID-19

Severe acute respiratory syndrome coronavirus 2 (SARS-CoV2) infection is the cause of COVID-19 [6]. SARS-CoV-2 uses a spike protein S1 to enable the attachment of the virion to the cell membrane of the host and then mRNA encoding several other proteins, by interacting with the host angiotensin-converting enzyme 2 (ACE2) receptors [13]. Besides, the SARS-CoV2 spike protein has a 10–20-fold binding affinity to the human ACE2 receptor higher than that of the SARS-CoV the causative agent of SARS disease which explains the high contiguity of the SARS-CoV2 [14]. ACE2 receptors are expressed principally in type II pneumocytes which are considered the predominant gateway for SARS-CoV-2 entry [6]. ACE2 receptors and transmembrane protease serine 2 (TMPRSS2) are expressed in different body systems like the gastrointestinal, urinary, and reproductive organs [3, 15]. It has been found that ACE2 receptors are highly expressed in the cardiovascular tissue including endothelial cells, cardiac myocytes, fibroblasts, and smooth muscle cells [6, 16, 17]. ACE2 receptors were presumed to counteract the effects of angiotensin II in cases associated with the renin–angiotensin system (RAS) overactivity, such as hypertension, atherosclerosis, and congestive heart failure (HF). Thus, according to this, there is a direct relationship between COVID-19 and increased deaths and morbidity from cardiovascular disease (CVD) [6]. Furthermore, higher expression of ACE2 may facilitate penetration of the virus into the cell, prolong the virus life cycle, and enhance virus replication [18]. The exact mechanism of cardiovascular involvement in COVID-19 is still unclear, and several mechanisms have been proposed [3, 19]; it may be a result of direct or indirect injury [3]. Direct myocardial injury may result from direct viral toxicity through binding to ACE2 receptors present in the cardiac muscle [3, 19]. Pathological examination of COVID-19 autopsies confirmed the presence of SARS-CoV2 viral copies in the myocardium as well as other organs including the endothelial cells of the venous and arterial system (orgamotropism) [20–22]. The spread of infection to the myocardium and other organs is still unclear and thought to be through hematogenous spread (viremia) [20].

Indirect cardiac injury may be attributed to immune system dysregulation with the release of cytokines, thrombo-inflammation, microcirculation dysfunction, endothelial cell damage, and dysregulation of the renin–angiotensin–aldosterone system (RAAS) [3]. Cardiac injury can result from the abnormal systemic inflammatory response with exaggerated secretion of inflammatory mediators (cytokine storm). These inflammatory markers include: interleukin (IL-) 1B, IL-2, IL-6, tumor necrosis factor-alpha (TNF-α) [23, 24], monocyte chemoattractant protein-1 (MCP-1) [23]. This cytokine storm is enhanced in the presence of aberrant T helper cells immune response and the increased intracellular calcium secondary to hypoxia. The net result is cardiac myocyte apoptosis [23]. Inflammatory cell infiltration of the myocardium was detected in several autopsy studies [25, 26]. Myocarditis is a significant factor in patients with acute HF [11].

A preexisting cardiovascular disease is a risk factor for severe COVID-19 due to increased expression of ACE2 receptors [27, 28]. Increased pulmonary pressure in respiratory failure and ARDS may result in right ventricular dysfunction [29, 30]. Pulmonary thromboembolism can be considered as another important factor [31, 32].

Physiologic stress with hypoxemia is a contributing factor that may result in myocardial ischemia, tachycardia, and several cardiovascular adaptations [33–35].

Partly, arrhythmias may be attributable to hypoxia, inflammatory stress, and abnormal metabolism in SARS-CoV-2-infected patients with or without preexisting CVD [36]. Various types of arrhythmias may result as a side effect of drugs used in treatment COVID-19 [11]. Several studies reported arrhythmias in cases with severe COVID-19 pneumonia due to gas exchange abnormalities secondary to hypoxemia [37, 38]. Dysrhythmia may further complicate the disease course [11].

Hypoxemia is associated with a significant reduction in the energy supply and increases anaerobic metabolism resulting in intracellular acidosis and excess oxygen free radicals that destroy the cell membrane [37, 38]. As a consequence of systemic infection and hypoxia, cardiometabolic demand is elevated, and in the presence of inadequate supply, myocardial oxygen supply/demand mismatch occurs leading to myocardial damage [39].

Malavazos et al. proposed that the epicardial adipose tissue (EAT) contributes to the development of myocarditis. EAT has a direct anatomical and functional relationship to the myocardium, and both share the same microcirculation. During SARS-CoV2 infection, EAT is highly infiltrated with macrophages and cytokines like IL-6 which can reach the pericardium and myocardium directly via the vasa vasorum or through paracrine pathways [40].

The hypercoagulable state was a prominent finding in COVID-19 patients in several reports. Laboratory findings in these patients included: high serum ferritin [7]. High levels of D-dimers, fibrinogen, CRP decide prolonged prothrombin time (PT). Fibrinogen levels may decrease in advanced disease. Leukopenia, lymphopenia, and thrombocytopenia are also common features [41, 42].

Increased morbidity and mortality in COVID-19 may be attributed to several risk factors including male gender, old age, obesity, hypertension, diabetes, and established cardiovascular and cerebrovascular disease [7, 43]. Poor outcome in these patients can be explained by uncontrolled viral replication and prolonged pro-inflammatory response as a result of the excess secretion of type 2 cytokines and the age-related T-cell and B-cell dysfunction [43]. Further, older age and male sex were associated with an increased level of expression of ACE2. The upregulation of ACE2 has been reported as significantly high in patients with diabetes mellitus (DM) and hypertension who receive ACE inhibitors (ACEIs) or angiotensin receptor blockers (ARBs) [44, 45]. These results led to the assumption that those patients are more susceptible to infection with SARS-CoV2. ACE2 polymorphisms are more likely to be a hereditary risk of developing infection with COVID-19 [46]. VTE in COVID-19 may result from abnormal coagulation, systemic inflammatory response, multiorgan dysfunction, critical illness [47], hormonal therapy, prolonged bed rest, and immobilization [48]. Prolonged hospitalization with decreased mobility and severe respiratory disease are also risk factors for VTE [49]. Even lockdown has been associated with increased risk of CVD [50].

### Cardiovascular comorbidities in COVID-19

Comorbid illnesses are prevalent in severe COVID-19 cases. Cardiovascular comorbidities were reported to be a key player due to its effect on prognosis. It has been reported that CVD increased the case-fatality rate by 10.5%, compared to 7.3%, 6.3%, 6%, and 5.6% for DM, chronic obstructive pulmonary disease (COPD), hypertension, and cancers, respectively [51]. Similar behavior was noted for MERS-CoV and SARS [52, 53]. Patients with CVD have increased susceptibility to infection with COVID-19 and are prone to serious and complicated symptoms [54].

### Cardiovascular complications COVID-19

Management of myocardial dysfunction during the current pandemic is a great challenge. Several factors are to be considered including the challenges to perform cardiac imaging, hemodynamic assessment, and endomyocardial biopsies on the one side and the hazard of viral infection to patients and healthcare professionals (HCP) as well as contamination of healthcare facilities on the other side [55, 56].

Cardiac involvement may occur several days after influenza-like symptoms [57]. The frequency of cardiovascular symptoms in COVID-19 is still unknown. Most patients develop gradual deterioration. The median interval between the onset of symptoms and admission to ICU ranged between 9 and 10 days [58]. Chest pain or tightness with or without breathlessness is the most common symptom, and pain is usually poorly localized. Myocardial infarction type II may be suspected if chest pain and ECG changes are associated with altered biomarkers[35]. Typical symptoms related to myocardial ischemia are more reported in ACS patients [35].

**Fig. 2:**
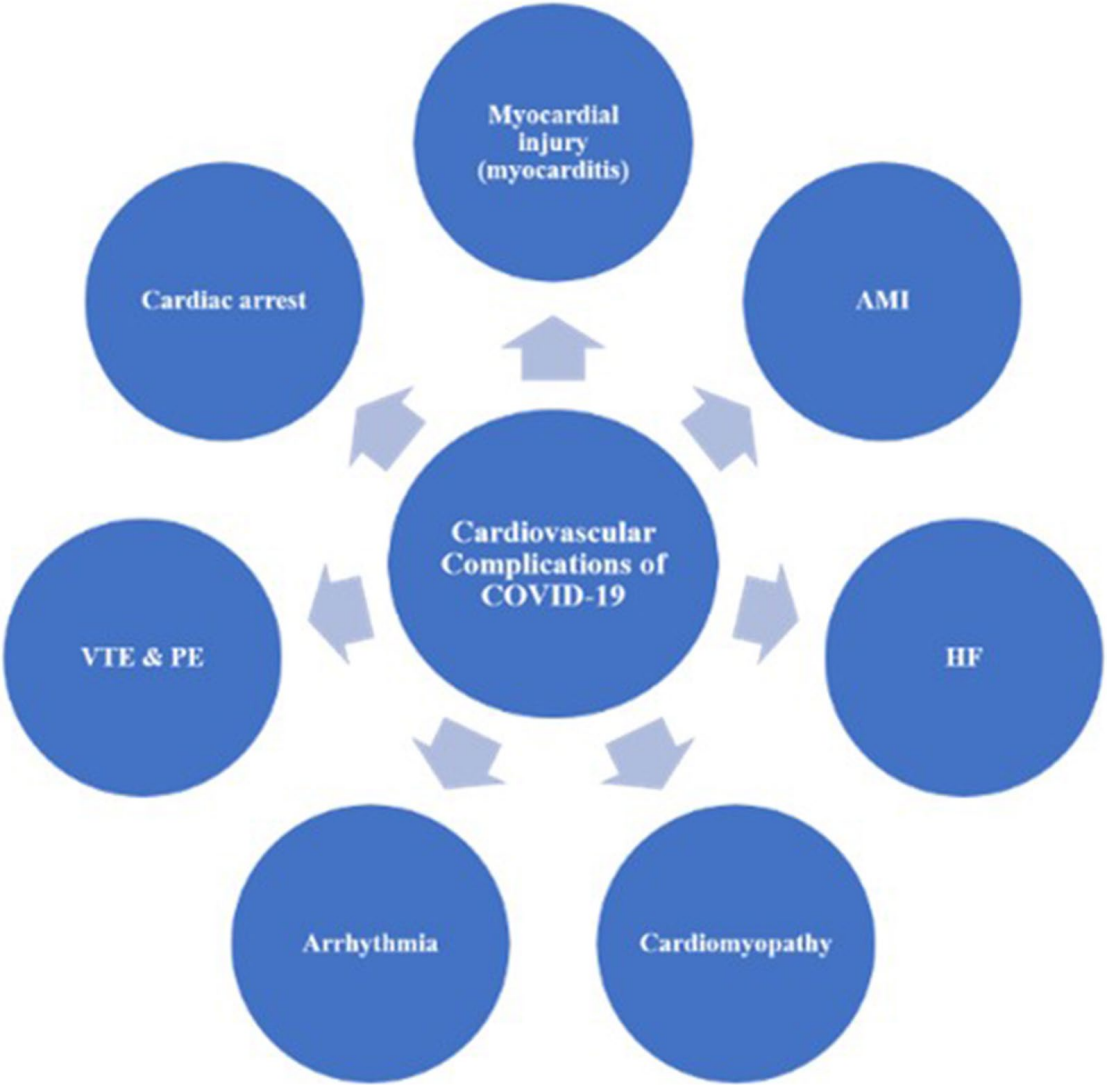
Spectrum of cardiovascular complications in COVID-19. A wide spectrum of cardiovascular complications in COVID-19 is reported. AMI: acute myocardial infarction; HF: hear failure; VTE: venous thromboembolism; PE: pulmonary embolism

A large spectrum of CV complications can develop in COVID-19 patients (Fig. 2). Patients with COVID-19 may present with various cardiovascular conditions as cardiomyopathy (either stress or non-ischemic), ACS, AMI, myocarditis simulating a stable ST-segment elevation myocardial infarction (STEMI), myocardial injury without evidence of Type I or Type II AMI [51, 59], pulmonary thromboembolism, acute corpulmonale, isolated right ventricular failure, and cardiogenic shock (CS) [3, 31, 32], cardiac arrhythmias [60], and heart failure [59]. Critically ill patients may develop pneumonia, ARDS, multi-organ dysfunction, hemodynamic instability, and several cardiovascular complications [51, 59].

### Acute myocardial injury

Myocardial injury is defined as a remarkable rise in troponin levels. It may occur due to ischemic or non-ischemic myocardial injury as myocarditis [61]. Acute myocardial injury evidenced by elevated cardiac biomarkers, characteristic ECG, and new imaging features of impaired cardiac function was recognized among COVID-19 cases and is suspected to occur in patients who had hypoxia or ARDS [6, 43]. Myocarditis may be the initial presentation in some cases [62].

### Acute myocardial infarction (AMI)

Increased incidence of AMI has been reported with several respiratory viruses including influenza and other coronavirus species [63]. Severe inflammation and hypercoagulation increase the likelihood of AMI in patients with COVID-19 [64]. Acute myocardial infarction (AMI) is an urgent situation that requires emergency management. Infection with SARS-COV-2 greatly affected AMI diagnosis and treatment [64]. SARS-CoV-2 infection can result in several cardiovascular manifestations even without respiratory symptoms or constitutional manifestations like fever and bony aches [57].

### Heart failure (HF) and cardiomyopathy

Heart failure may be the principal clinical feature of COVID-19 infection [10, 62]. The frequency of HF in COVID-19 patients varies from 0.4 to 43% in previous reports [65]. Myocardial damage and HF accounted for nearly 40% of deaths in critically hospitalized patients [59].

### Cardiac arrhythmia

Arrhythmias may associate or complicate COVID-19 infection [10]. Palpitations have been reported by some patients, and sinus tachycardia was the most common type of dysrhythmia observed [66]. Hypoxemia in COVID‐19 patients can trigger AF, particularly in the elderly [67]. A cohort study in China demonstrated that cardiac dysrhythmia was present in 16.7% of hospitalized patients with COVID-19. The frequency among ICU patients was 44.4% compared to 6.9% for non-ICU patients [60]. Several types of cardiac arrhythmias were also reported including ventricular arrhythmias, new-onset atrial fibrillation (AF), and heart block [60].

### Venous thromboembolism (VTE) and pulmonary embolism

Patients suffering from COVID-19 infection are also at high danger of developing VTE. Thrombotic events were more probable in critically ill COVID-19 patients despite the use of proper anticoagulation [48]. Cui et al. reported 25% of hospitalized patients with COVID-19 had DVT of lower extremities. No anticoagulation had been administered to those patients [41]. Bompard et al. [68] and Grillet et al. [69] observed PE in nearly one-fourth of patients with COVID-19 pneumonia who underwent contrast-enhanced CT pulmonary angiography. Also, autopsies from patients who died from COVID-19 showed pulmonary microthrombi [70]. Thrombosis in COVID-19 may be attributed to hypercoagulable state associated with inflammatory process resulting in endothelial activation and dysfunction, platelet activation leading activation of coagulation cascade. Abnormalities in renin–angiotensin system associated with COVID-19 result in vasoconstriction and cytokine release which promote thrombosis. moreover, the tissue damage due to cytokine storm and weak immune system increase the risk of thrombosis [71].

### Cardiac arrest

Researchers reported an increased incidence of out-of-hospital cardiac arrest during the COVID-19 pandemic in 2020 compared to the same period during 2019 [12]. Several reports of cardiogenic shock [62] or sudden cardiac arrest in COVID-19 patients had been published [72].

## Diagnostic tools

### Electrocardiogram (ECG) in COVID-19 patients

No specific ECG changes are described in COVID-19 patients. This may be attributed to the subtle myocardial involvement associated with infection [57]. So, the same ECG diagnostic criteria in the general population are applied for COVID-19 patients [60]. ST-segment elevation was reported in myocarditis [57]. Arrhythmias were reported in various percentages of patients especially those who were admitted to ICU [60]. Prolonged QT (> 500 ms) was detected at the time of hospitalization in 6.1% of patients with COVID-19 in a multicenter research [73]. New ECG changes may indicate cardiac complications especially in those with elevated biomarkers [43]. ECG should be done to record the patient’s baseline QT interval before receiving drugs that may prolong QT interval [74].

### Imaging

Any elective cardiac imaging should be delayed until the patient is considered non-infectious [75]. Transthoracic echocardiography is the recommended investigation for hospitalized patients with HF, suspected MI, new ECG changes, or newly diagnosed cardiomegaly in chest imaging. Cardiac CT and MRI are helpful; however, these will lead to unwanted exposure to the disease burden [57, 76]. As Well, point-of-care echocardiography may be employed to assess regional myocardial contractile function and to guide decisions about invasive assessment [77].

### Markers of cardiac injury

The elevated level of cardiac biomarkers in COVID-19 is a prominent feature that indicates cardiac involvement and is associated with poor clinical outcome [37, 78]. Elevated biomarkers of cardiac injury were detected in a significant number of admitted cases with COVID-19. The prevalence is more in those with pre-existing cardiovascular disease [23, 43, 78]. High-sensitivity cardiac troponin I [hs-cTnI] level was elevated in COVID-19 patients (7.2% overall cases and 22% of critical care patients). Elevated troponin level was also detected in nearly 12% of COVID-19 patients with no previous history of CVD and was associated with poor prognosis [6, 43]. A significant positive linear correlation between plasma TnT levels and plasma high sensitivity CRP levels indicates the close association between myocardial injury and the inflammatory process [23].

The levels of the biomarker of hemodynamic myocardial stress and HF which is B-Type Natriuretic Peptide/N-Terminal B-Type Natriuretic Peptide (BNP/NT-proBNP) are commonly elevated among patients with severe inflammatory disorders including respiratory ones. The high levels of BNP/NT-proBNP in a patient with SARS-CoV-2 infection may suggest the presence/magnitude of pre-existing cardiac condition, especially the acute hemodynamic stress and/or the right ventricular strain linked to COVID-19 [78].

High levels of inflammatory markers of cardiac injury have been noted to be associated with electrocardiographic abnormalities in COVID-19 [23].

Significant coagulation abnormalities exist in hospitalized patients with severe COVID-19 illness [48]. D‐dimer and fibrin degradation product (FDP) levels were significantly elevated in the non-surviving COVID-19 patients. Those who had high FDP fulfilled the diagnostic clinical parameters for disseminated intravascular coagulation [42]. A D-dimer level greater than 1 μg/mL during hospitalization was correlated with lethal outcomes [42, 43].

Furthermore, an abnormal immune response may be detected in COVID-19 patients as high expression of ACE2 receptors in the SARS-CoV-2 infected cells, high levels of IL-1, IL-6, IL-8, and IL-10 [18]. High levels of IL-6, LDH, ferritin, and D-dimer were all linked to myocardial injury [19].

### Implications on diagnosis

The differential diagnosis of cardiovascular manifestations in presumed or evident COVID-19 infection is complicated and tricky. Various respiratory symptoms as shortness of breathing and chest pain are also cardiac symptoms. Dyspnea may also precede or associate other cardiac manifestations. Soon, a precise and quick determination of cardiovascular problems, in this case, is vital [35]. Clinicians should have a high index of clinical suspension of cardiac injury in the setting of COVID-19 pandemic. Prompt clinical evaluation is mandatory if cardiovascular involvement is suspected [46]. In individuals with acute illness who had recent cardiac symptoms, a thorough clinical examination and laboratory investigations, including troponin levels, are warranted. This will ensure appropriate recognition and prompt isolation of suspected cases and decrease the spread of infection [57].

### Implications on case management

No specific treatment for COVID-19 has been reported or approved until the time of writing this review. The treatment is symptomatic, and some drugs were used due to their potential antiviral activity against different viruses rather than COVID-19.

It is important to protect the cardiovascular system in COVID-19 cases to prevent chronic cardiac damage [7].

The increased incidence of thromboembolic events in COVID-19 cases reported in several studies emphasized the benefits of using anticoagulants as a line of treatment in these cases. Low molecular weight heparin (LMWH) is commonly used in hospital settings and was associated with decreased morbidity and mortality [79]. Thromboprophylaxis was used only in those with a high risk of thrombosis [80]. However, some researchers suggested the administration of thromboprophylaxis in hospitalized COVID-19 to avoid the development of VTE and were found to be associated with better outcomes [81, 82].

The potential impact of acetylsalicylic acid (ASA) therapy has been questioned and still under investigation [83]. Nevertheless, the low doses of ASA have a minimal anti-inflammatory effect [84]. Those who use acetylsalicylic acid for long-term prevention of cardiovascular events are not advised to withhold it [85]. Nonsteroidal anti-inflammatory drugs (NSAIDs) could be a risk factor for the serious clinical presentation of COVID-19 [86]. The administration of NSAIDs may also hide some of the clinical manifestations leading to delayed diagnosis and treatment [85].

The acute treatment of arrhythmias and MI is similar to that in the general population. The same guidelines apply   with consideration of the safety of healthcare professionals (HCP) [87, 88]. Primary percutaneous coronary intervention is the ideal decision for STEMI [64, 89], and in case of absence of personal protective equipment (PPE), fibrinolytic therapy may be an appropriate option [89, 90]. COVID-19 patients with non-ST-segment elevation myocardial infarction (NSTEMI) and who are hemodynamically unstable are managed as STEMI patients. Selected cases with NSTEMI and COVID-19 can undergo conservative therapy [77].

Adjusted fluid therapy management is critical in COVID-19 patients to avoid cardiovascular complications especially in those with ARDS who are liable to right heart failure [36].

**Fig. 3:**
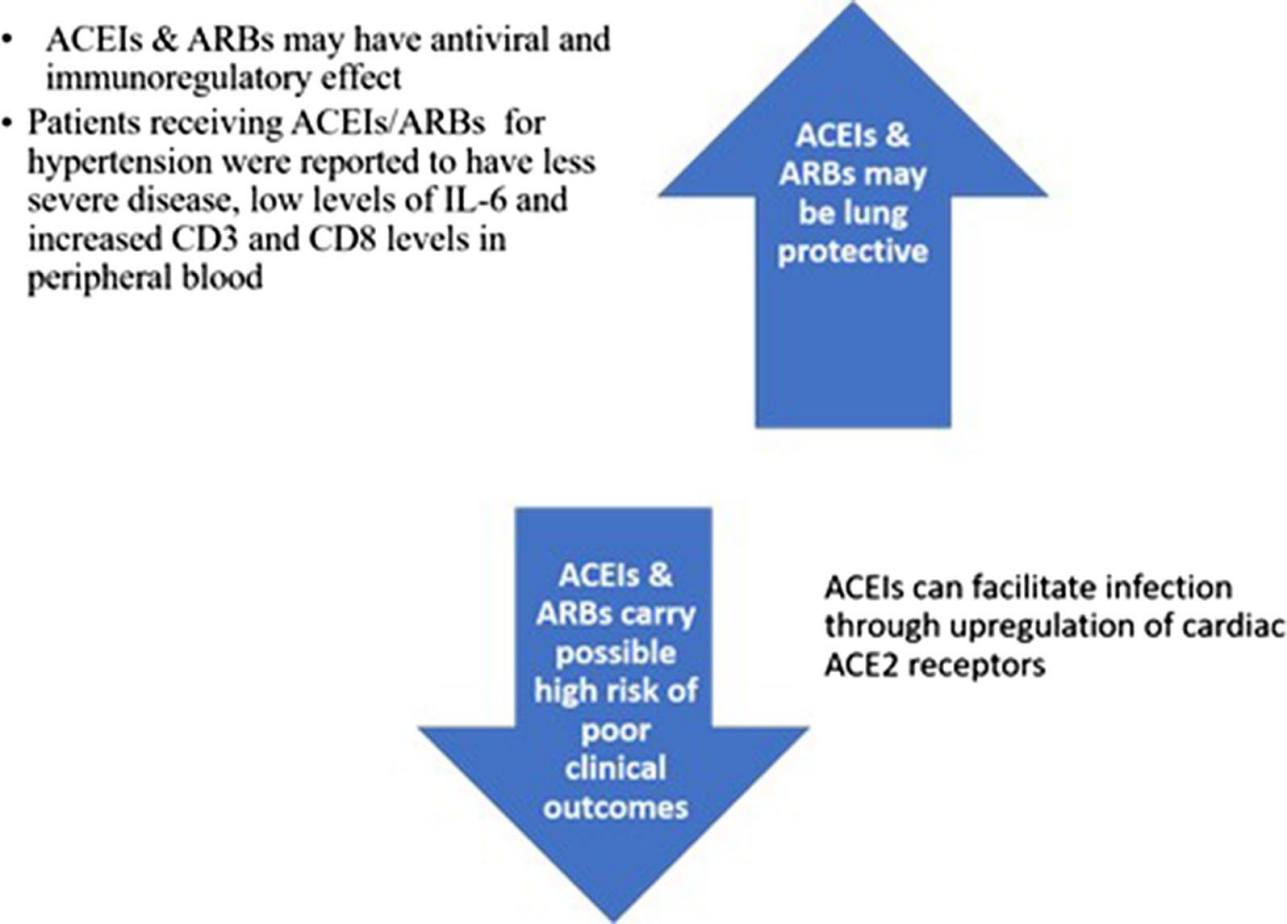
Controverse about the role of ACEIs & ARBs in COVID-19: There is controverse about the use of ACEIs & ARBs in COVID-19

There is an argument about the use of some drugs used for the treatment of CV diseases, especially RAS blocking drugs as ACEIs and angiotensin receptors blocking agents (ARBs) in patients with COVID-19 [91, 92] (Fig. 3), and the mechanism of involvement of these drugs in COVID-19 is still unclear involving several mediators, neural and chemical mechanisms. Some researchers suggested withholding ACEIs or ARBs in COVID-19 patients and may be replaced by calcium channel blockers [46]. Their opinion is based on limited data supposing that ACEIs lead to upregulation of cardiac ACE2 receptors [93]. Controversy, more recent human studies reported no relationship between the use of these drugs and the severity, complications, and risk of death from COVID-19 after adjustment for other confounders [94–97]. Besides it may have immune-modulating effect [98–102], some studies reported no effect of ACEIs or ARBs on ACE receptors present in respiratory epithelium indicating no risk of increased susceptibility to COVID-19 infection [103]. At the current time, no evidence exists to support one of the two opposing opinions [91, 92]. So, discontinuation of RAAS in the setting of COVID-19 infection is not recommended [104, 105] as these drugs are key elements in the management of some cases [93]. Treatment of hypertension in COVID-19 suspected or infected patients should continue to follow the current guidelines [105].

Statins have potential value in COVID-19. It may reduce risk of severe disease and hospitalization due to anti-inflammatory and immune modulating properties as well as antilipid effect as lipid metabolism contributes to pathogenesis of SARS-CoV-2 infection [106]. On the other hand, statins may lead to upregulation of ACE2 [107]. A recent metaanalysis concluded that statins could be of benefit in COVID-19 and no evidence against its use. However, randomized controlled trials of good quality are warranted to validate this conclusion [108].

Severe rhabdomyolysis or increased liver enzymes have been reported in some cases with COVID-19, and in such cases, it may be wise to stop statin therapy till the condition improves [82, 109].

Some drugs that are used in treatment protocols of COVID-19 may have some adverse events on the CVS or interact with drugs used for the treatment of CV conditions (Table 1). These drugs include lopinavir/ritonavir, remdesivir, chloroquine, and hydroxychloroquine.

**Table 1:** Adverse events of some drugs used in treatment COVID-19 on cardiovascular system

Drug	Adverse CV effects
Lopinavir/ritonavir [36]	ECG changes including prolonged PR and QT intervals Interactions with some anticoagulants especially CYP3A mediated drugs
Remdesivir [109, 110]	Hypotension
Chloroquine and hydroxychloroquine [110, 111]	Cardiac arrhythmias either atrial or ventricular, ventricular fibrillation Heart Failure, Atrioventricular block, bundle branch block Cardiomyopathy Hypotension ECG changes including flat or inverted T wave, prolonged QT interval, wide QRS complex

ECG: electrocardiogram

Lopinavir/ritonavir may prolong PR and QT intervals in ECG, especially in the presence of baseline abnormalities like (long QT), risk of conduction abnormalities, and those receiving drugs that prolong QT interval [36]. Lopinavir/ritonavir has drug interactions with some anticoagulants, especially CYP3A-mediated drugs like rivaroxaban and apixaban. Dose reduction or avoidance of this group of drugs is recommended if lopinavir/ritonavir is used for treatment [36]. Remdesivir resulted in hypotension in some cases [109, 110]. Chloroquine and hydroxychloroquine have cardiovascular side effects including cardiac arrhythmias either atrial or ventricular, HF, conduction abnormalities, cardiomyopathy, hypotension, and ECG changes including flat or inverted T wave, prolonged QT interval, wide QRS complex [110, 111].

### Infection control measures

**Fig. 4:**
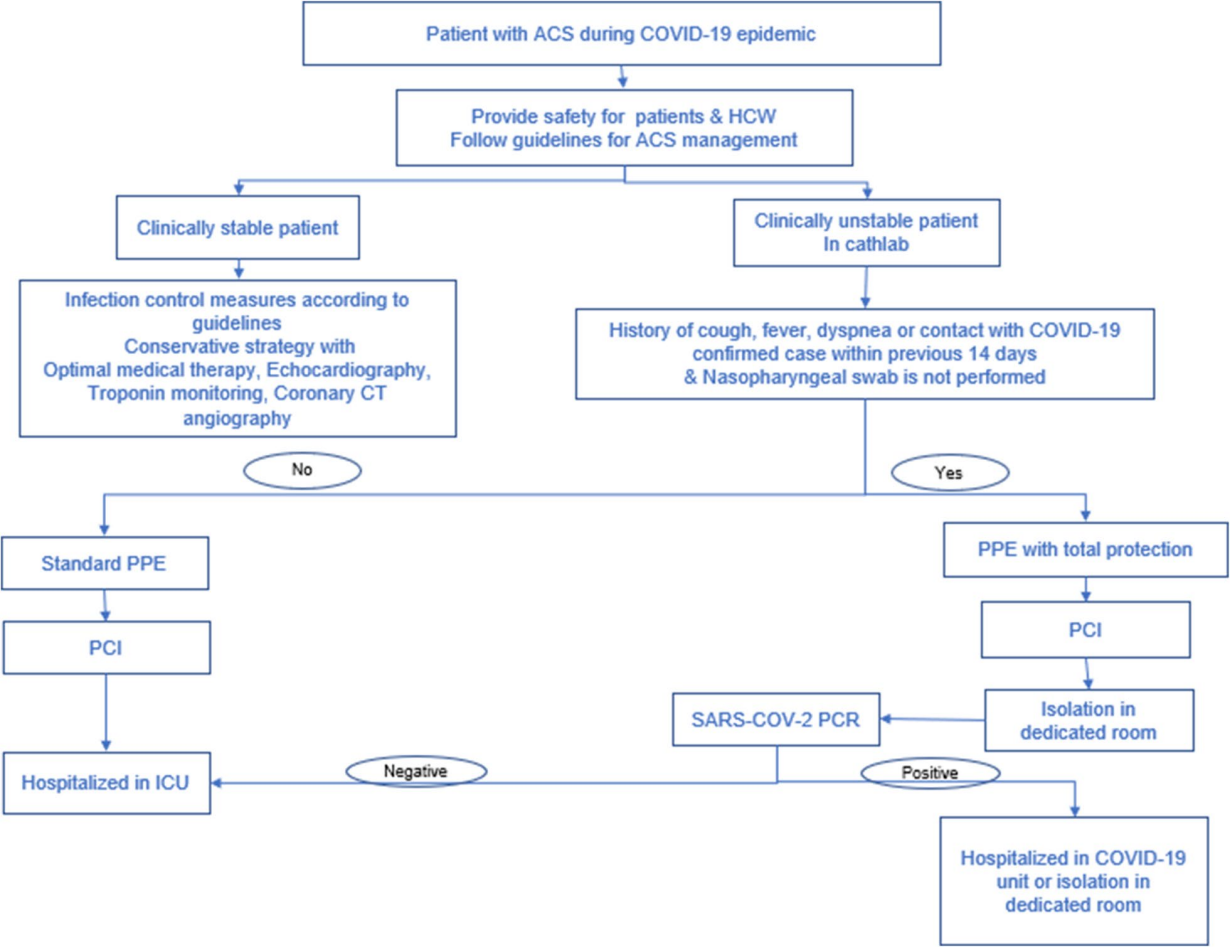
Algorythm for management of acute coronay syndrome during COVID-19 epidemic. ACS: acute coronay Syndrome, ICU: intensive care unit, PPE: personal protective equipment, HCW: healthcare workers

For infection control purposes during the COVID-19 pandemic, diagnostic testing before any intervention is highly recommended [77]. Besides, in the absence of the SARS-CoV-2 testing results, they should be managed as if they are COVID-19 positive [88]. All HCP exposed to suspected/confirmed COVID-19 cases should adhere to infection control measures and wear appropriate PPE [11]. All non-urgent and/or elective investigational and/or interventional procedures should be postponed till resolution of COVID-19 infection to avoid unnecessary exposure of HCP to the disease burden taking into consideration the balance between staff exposure and patient benefit [11, 57, 75–77]. In emergency cases where cardiac catheterization is indicated, it should be executed in a devoted COVID-19 catheterization laboratory if available [11] (Fig. 4). Careful decontamination of the cardiac intervention laboratories following the procedure is recommended. Rapid discharge is recommended after revascularization of patients with primary NSTEMI to reduce patient exposure within the hospital [77].

### Implications on public health

Public health focuses on discovering and modernizing the environmental factors associated with diseases. The role of public health is on the individual or at the community level [112].

### Short-term effects and dilemma of resource allocation

During the current pandemic, it is impossible to treat all patients, especially in critical care settings [113]. The need for rearrangement of healthcare services during the COVID-19 pandemic raised several issues regarding the prioritization of cardiovascular procedures to postpone procedures which are not urgent. An important issue is to put a strategy that guarantees that patients with emergency cardiac conditions will receive standard of care and had timely access to emergency services [88, 114]. Allocation of HCR or redistribution of healthcare services may intervene with decision-making. Resource allocation decisions often concentrate on the instant payoff for risk reduction from a specific disease. However, resource allocation decisions can impact the infrastructure required to respond over time to health risks [115]. So, we have faced by emerging ethical dilemmas: How healthcare resources (HCR) can be allocated?, how to put the HCR allocation guidelines? what do we rely on in the setting of the posterization for emergency health services? How many HCR allocation decisions will be carried out and how this will affect the delivery and distribution of health benefits? Which strategy will be used to deal with competing health policy priorities? [113, 116]. Shall we allocate HCR to those who can be saved or those with the worst clinical situation? And why? [115].

Treatment strategies with controversial value will be a great challenge for HCR allocation decisions as some will see these strategies as life-saving and others see it of unproven efficacy and un-necessary action [117]. So, the two opposing public health values stewardship and compassion will appear. Whatever the best decision will be, most public health readers would like to present themselves to the community as dedicated to saving lives rather than conserving resources [115].

Usually, Medicine tells us what we can do; however, ethics tells us what we should do. Principles of medical ethics include: autonomy, beneficence, non-maleficence, and justice [118]. Generally, physicians/cardiologists follow one of three mechanisms: (i) external constraints, not admitting a patient from the ER who needs intensive care as all available beds are full and no patient can be safely discharged (e.g., all on ventilators). Or a patient who may need an emergency cardiac catheterization for an emergency coronary insufficiency but, the hospital’s only interventional cardiologist is occupied in a lengthy procedure [119]. The second mechanism (ii) clinical guidelines set by the organization that may control the decision of clinicians when dealing with an emergency during pandemics, third mechanism is the (iii) clinical judgment which depends on the cardiologist experience; how to determine among competing patients who will receive the last bed or ventilator in the ICU. How to divide one’s time among all of the patients in the ER or canceling surgery for a patient with the three-vessel disease and with “stable” angina [119].

Most guidelines and clinical practice procedures make prioritization of patients with chronic cardiac conditions like chronic coronary syndrome based on risk stratification [88, 114]. Initiation of invasive procedures for the emergency cardiac conditions like STEMI may be limited due to the high risk of infection and the need for prolonged disinfection after the procedures, and less effective procedures can be applied [11]. These modifications of optimal care may result in increased cardiovascular-related complications and deaths [120]. And access to emergency care may be negatively affected by the redistribution of healthcare resources and delayed arrival to emergency rooms as most patients are frightened from catching infection [11].

### Long-term effects

The way of life and mental wellbeing sequel of COVID-19 may increment the load of CVD. Lockdown and social distancing promote sedentary behavior, sudden alterations in lifestyle, and unhealthy diets [120]. The physical inactivity has deleterious effects including ectopic fat distribution, metabolic imbalance, impaired cardiorespiratory function, osteoporosis, weakness of lower extremity muscles [121, 122], hyperinsulinemia and increased insulin resistance, significant stress and anxiety, worsening depression, and loneliness [120, 123, 124]. The net result will be increased incidence of cardiovascular events, limited access to healthcare services for preventive measures, and overall increased healthcare expenditure [120]. This was very evident as a substantial upsurge in the occurrence of stress cardiomyopathy during the COVID-19 pandemic. None of these patients was found to have COVID-19 disease suggesting indirect, psychological, social, and economic pandemic-related stress mechanisms behind the disease process [125].

### Implications on public health

COVID-19 pandemic has several immediate and long-term cardiovascular complications. Cardiovascular diseases have deleterious effects on clinical outcomes. Overall cardiovascular health is affected by the COVID-19 pandemic either directly or indirectly. Clinicians should have a high index of suspension and be very cautious in drug selection during the management of COVID-19 cases. Cardiovascular emergencies in COVID-19 patients are managed similar to the general population with appropriate considerations to prevent transmission of infection.

## Conclusions

COVID-19 is associated with negative consequences on the cardiovascular system. Meanwhile, preexisting cardiovascular complications are associated with poor prognosis. Knowing this fact should help improve local strategies set for management of cardiac problems during the COVID-19 pandemic.

## Abbreviations

ACE2: Angiotensin-2
ACEIs: Angiotensin-converting enzyme inhibitors
ACS: Acute coronary syndrome
AF: Atrial fibrillation
AMI: Acute myocardial infarction
ARBs: Angiotensin receptors blocking agents
ARDS: Acute respiratory distress syndrome
BNP: Brain natriuretic peptide
COPD: Chronic obstructive pulmonary disease
COVID-19: Coronavirus disease-2019
CRP: C-reactive protein
CS: Cardiogenic shock
CV: Cardiovascular
DM: Diabetes mellitus
ECG: Electrocardiography
FDP: Fibrin degradation product
HCP: Healthcare professionals
HCR: Healthcare resources
HF: Heart failure
hs-cTnI: High-sensitivity cardiac troponin I
ICU: Intensive care unit
IL: Interleukin
LMWH: Low molecular weight heparin
NSAIDs: Nonsteroidal anti-inflammatory drugs
NSTEMI: Non-ST-segment elevation myocardial infarction
NT-proBNP: N-Terminal B-type natriuretic peptide
PE: Pulmonary embolism
PPE: Personal protective equipment
RAS: Renin–angiotensin system
SARS-CoV-2: Severe acute respiratory syndrome coronavirus-2
STEMI: ST-segment elevation myocardial infarction
VTE: Venous thromboembolism
